# Utilization of Emergency and Outpatient Mental Health Services by LGBTQ Populations

**DOI:** 10.7759/cureus.88913

**Published:** 2025-07-28

**Authors:** Kristen Frank-Dixon, Mandy L Maneval, Hendrik Marais, Shirley Hochhauser, Emily Kraft, Marshall Herbst, Sunmeet Singh, Jordan Law, Tian Guo, Brenna Lash

**Affiliations:** 1 Family Medicine, Geisinger Health System, Lewistown, USA; 2 Family Medicine, Family Practice Center, Mifflintown, USA; 3 Medicine and Pediatrics, Geisinger Health System, Danville, USA; 4 Phenomic Data Analytics, Geisinger Health System, Danville, USA; 5 Biostatistics, Geisinger Health System, Danville, USA; 6 Psychology, Geisinger Health System, Danville, USA

**Keywords:** emergency care setting, lgbtq, outpatient care, rural population, suicide and depression, underserved populations, utilization of services

## Abstract

Background

Multiple studies have shown high and increasing rates of depression, suicidality, and marginalization of the LGBTQ population. As value-based care is increasingly utilized for healthcare reimbursement, health systems are tasked to manage population-based health for whole populations, including underserved communities. This study examines how LGBTQ+ individuals interact with emergency departments (EDs) and outpatient clinics relative to the control population, specifically in terms of access and utilization for mental health care in rural and semi-rural settings.

Methods

We performed a retrospective, population-based study using five years of electronic health record (EHR) data from Geisinger Health System's data warehouse. Demographic sexual orientation and gender identity (SOGI) data, as well as ICD-10 diagnostic codes, were used to identify the study population. Inclusion criteria were (1) non-heterosexual orientation and/or non-cis gender identity and (2) at least one contact with the health system between April 2017 and March 2022 (n=10,727). Controls were age- and insurance-matched (n=21,448). Data were summarized and analyzed for statistical significance using SAS.

Results

Rates of depression were significantly elevated in both TGD (transgender and gender diverse) and LGB (lesbian, gay, and bisexual) groups compared to their respective control populations. This trend was seen in both the adult and minor categories. The LGBTQ group utilized both outpatient clinic services and the ED for crisis care at higher rates when compared to the control population.

Conclusions

We conclude that despite greater utilization of outpatient services, the LGBTQ population still utilizes the ED more often for mental health crisis care than controls. These findings suggest that outpatient care may fall short in addressing the underlying mental health needs of LGBTQ individuals. This may be attributable to limited access to LGBTQ-informed care in the outpatient setting.

## Introduction

The number of individuals identifying as LGBTQ has been steadily increasing. A 2023 CDC survey of 17,508 U.S. students included in the Youth Risk Behavior Surveillance System found that the number of students identifying as LGBTQ has increased from 11% in 2015 to 26% in 2021 [1]. Similarly, the number and percentage of Americans who identify as LGBTQ have increased from 3.5% in 2012 to 7.6% in 2023 ​[2]​. In healthcare settings, this represents a significant portion of the patient base.

Multiple studies have identified high rates of mental health disorders, including depression and suicidality, among LGBTQ populations. A 2019 study used a national quantitative cross-sectional survey of 25,000 LGBTQ youth between the ages of 13 and 24 in the US [3]. Depressed mood, defined by feeling sad or hopeless for at least two weeks in the past year, was seen in 71% of LGBTQ study participants. Suicidality was seen in 39%, and 18% had attempted suicide. Startling rates were seen in transgender and non-binary youth, with 83% reporting depressed mood and 54% and 29% having suicidality or suicide attempt, respectively. Transgender males reported the highest rates of mental health issues across all groups examined, 86% reporting depression, 62% seriously considering suicide, and 35% having attempted suicide. This increased prevalence of serious mental health disorders remained after adjusting for age, family income, and race/ethnicity [3]. A recent study examined the impact of state-level anti-transgender laws on mental health among transgender youth and found an increase in past-year suicide attempts ranging from 7% to 72% [4]. It seems clear that this is a population at risk.

Published data also suggest a lack of access to LGBTQ-affirming mental health care for this population.  Prior research has identified gaps in mental health care services for transgender and gender diverse (TGD) populations, which include the lack of culturally responsive and gender-affirming providers, affordability issues, and stigma and fear of mistreatment in care settings [5]. Access to clinicians with knowledge of the specific needs of LGBTQ patients has been found to be a barrier, and more so for gender non-binary patients. A national transgender survey reported only 2.8% of respondents felt their routine provider knew “almost everything” about their gender-related care, while 19.4% felt their provider knew “almost nothing” [5]. This disparity in mental health services may be even more pronounced in rural areas, where access to care is even more limited. A 2022 cross-sectional survey, including self-reported mental health and mental health care needs, of 241 TGD adults from 25 counties from the Northeastern U.S. (69% lived in rural areas) showed that 51% of respondents utilized at least one gender-affirming mental health service, including psychotherapy and psychopharmacology [6].

A systematic review concluded that LGBTQ people have been found to experience inequities across healthcare systems resulting from cis-heteronormative attitudes and practices in health care systems. These included having assumptions made about their sexual practices, experiencing negative reactions from their healthcare provider after coming out, and in the transgender population specifically, reports of healthcare providers refusing to respect preferred pronouns or using deadnames [7]. Health care discrimination and avoidance of health care due to fear of discrimination were reported in 29.3% and 21.6%, respectively [5]​. 

The primary objective of this study is to examine patterns of mental health outcomes and healthcare utilization among LGBTQ patients within a regional health system, with a focus on identifying disparities in depression, suicidality, and crisis care utilization. The secondary objectives are (1) to identify specific health system touchpoints where mental health outcomes and quality of care for LGBTQ patients can be measured and improved and (2) to explore opportunities for strengthening outpatient mental health services to reduce unnecessary emergency department (ED) utilization among LGBTQ individuals.

## Materials and methods

This is a retrospective study using five years of aggregate data from the Geisinger Health System data warehouse. A comparison between patients in different LGBTQ groups was performed, utilizing demographic sexual orientation and gender identity (SOGI) data as well as ICD-10 diagnostic codes.  Inclusion criteria included (1) non-cis gender and/or non-heterosexual orientation as determined by self-reported "Sexual Orientation and Gender Identity" forms in the electronic health record (EHR), mismatch between legal gender and reported gender identity, or from the ICD-10 codes F64 denoting transgender diagnoses. Diagnoses of major depression and suicidality were identified using ICD-10 codes applied in the patient records. All data was extracted and analyzed in aggregate. 

Gender identity was classified based on self-reported SOGI data in the EHR (categories: transmasculine, transfeminine, and non-binary). In this study, the term "transgender" refers to individuals whose gender identity does not align with the sex they were assigned at birth. "Transmasculine" describes individuals who were assigned female at birth but identify as male or predominantly masculine. In contrast, "transfeminine" refers to individuals assigned male at birth who identify as female or predominantly feminine.

Controls were age- and insurance-matched (n=21,448). Frequencies and percentages were used to describe categorical variables. Mean and standard deviation were used for normally distributed continuous variables. Median and interquartile range were used for non-normally distributed continuous variables. Comparison between the groups was performed using chi-square for categorical variables and the Wilcoxon rank test for continuous variables. P-values less than 0.05 are considered significant. All analyses were performed using SAS Enterprise Guide (SAS Institute Inc., Cary, NC, USA). 

The study proposal was reviewed by Geisinger’s Institutional Review Board and determined to be exempt as the research does not include human subjects.

## Results

This study examined 10,727 minor and adult LGBTQ patients and 21,448 age- and insurance-matched controls (Table 1 and Table 2).

**Table 1:** LGB population overview To prevent redundancy, the LGB groupings are trans-exclusive. LGB, lesbian, gay, and bisexual

	Gay # (%)	Lesbian # (%)	Bisexual # (%)	Other # (%)
All ages (n=8,684)	1,811 (21%)	1,662 (19%)	4,709 (54%)	502 (6%)
Adults (n=8,388)	1,792 (21%)	1,624 (19%)	4,504 (54%)	468 (6%)
Minors (n=296)	19 (6%)	38 (13%)	205 (69%)	34 (11%)

**Table 2:** TGD population overview Given the multiple potential methods for identifying the gender-specific transidentities, there is some minor overlap in some categories. TGD, transgender and gender diverse

	Total	Transmasculine	Transfeminine	Non-binary	Gender dysphoria diagnosis
All ages (n=2,043)	2,043	611 (30%)	369 (18%)	1,060 (52%)	1,243 (61%)
Adults (n=1,596)	1,596	466 (29%)	335 (21%)	793 (50%)	959 (60%)
Minors (n=447)	447	145 (32%)	34 (8%)	271 (61%)	284 (64%)

There are a greater number of individuals identifying transmasculine compared to transfeminine, and over half of the population has a non-binary gender identity, consistent with national data [8]. These differences are greater in the adolescent population compared to adults, which also correlates with national trends toward greater non-binary and transmasculine identities in younger populations [9].

This study observes significantly higher rates of depression and suicidality in the LGBTQ population; rates of depression were two- and threefold higher in the lesbian, gay, and bisexual (LGB) and TGD groups, respectively, compared to controls (Table 3 and Table 4). In minors, the differences were even more pronounced, with 59% of the transgender minor group having a diagnosis of major depression compared to 12.4% in the control group, and 8.5% having had an episode of suicidality resulting in a visit to the ED compared to 1.4% in the control group (p≤0.01). Note that this data only reflects patients who had at least one visit with the health system and does not include those who do not come for care, which likely underestimates the burden of depression and suicidality in the LGBTQ population.

**Table 3:** Diagnosis of major depression among adults and minors TGD, transgender and gender diverse; LGB, lesbian, gay, and bisexual

Group	Number (%)	P-value
Adult TGD (n=1,594)	806 (50.6%)	<0.01
Adult LGB (n=8,380)	3,272 (39.0%)	<0.01
Adult control (n=14,985)	2,410 (16.1%)	
Minors' TGD (n=447)	264 (59.1%)	<0.01
Minors' LGB (n=296)	123 (41.6%)	<0.01
Minors' control (n=6,461)	799 (12.4%)	

**Table 4:** ED utilization for suicidal ideation among adults and minors The number of patients who had at least one ED visit for suicidal ideation during the study timeframe. ED, emergency department; TGD, transgender and gender diverse; LGB, lesbian, gay, and bisexual

Group	Number (%) of patients with ED visit for suicidal ideation	P-value
TGD adult (n=1,594)	105 (6.6%)	<0.01
LGB adult (n=8,380)	229 (2.7%)	<0.01
Control adult (n=14,985)	145 (1.0%)	
TGD minor (n=447)	38 (8.5%)	<0.01
LGB minor (n=296)	13 (4.4%)	<0.01
Control minor (n=6,461)	89 (1.4%)	

Patients were determined to have depression or suicidality based on data extraction of ICD-10 codes for Major Depression and Suicidal Ideation in aggregate. It is noted that these data only include patients who had contact with the health system, which could lead to an underestimation of the true prevalence of depression and suicidality.

Outpatient utilization was assessed by measuring the total number of outpatient appointments completed and the percentage of appointments specifically addressing mental health concerns. The LGB and TGD groups not only had more visits overall, but also a greater percentage of these visits were made to address mental health concerns compared to controls (Table 5). Utilization of outpatient care, including mental health services, is found to be higher in the LGBTQ groups compared to controls.

**Table 5:** Outpatient visits for mental health in adults and minors Total number (%) of outpatient visits in any department where mental health diagnoses were addressed. TGD, transgender and gender diverse; LGB, lesbian, gay, and bisexual

Group	Total # visits	Avg # visits per year	Total number (%) of visits for mental health	P-value
TGD adult (n=1,368)	19,720	2.9	2,461 (12%)	<0.001
LGB adult (n=7,522)	1,33,997	3.6	6,434 (5%)	<0.01
Control adult (n=16,803)	1,68,979	2.0	4,908 (3%)	
TGD minor (n=812)	15,396	3.8	4,322 (28%)	<0.001
LGB minor (n=1,526)	18,422	2.4	3,453 (19%)	<0.001
Control minor (n=4,599)	34,537	1.5	2,900 (8%)	

TGD and LGB groups were compared to matched controls. When specifically examining mental health-related ED visits, TGD adults demonstrated a threefold higher rate than controls (Table 6). In contrast, while the LGB adult group showed increased overall ED utilization, the proportion of visits attributed to mental health reasons was relatively lower.

**Table 6:** ED visits for mental health in adults and minors ED, emergency department; TGD, transgender and gender diverse; LGB, lesbian, gay, and bisexual

Group	Total # ED visits	Avg # visits per year	Total number (%) of ED visits for mental health	P-value
TGD adult (n=1,594)	2,477	1.6	402 (16%)	<0.001
LGB adult (n=9,177)	14,622	1.6	649 (4%)	<0.01
Control adult (n=14,985)	20,314	1.4	1,027 (5%)	
TGD minor (n=447)	1,079	2.4	380 (35%)	<0.001
LGB minor (n=425)	1,492	3.5	246 (16%)	<0.001
Control minor (n=6,461)	3,081	0.5	364 (12%)	

Among minors, the TGD population again showed a threefold higher proportion of mental health-related ED visits compared to controls, whereas LGB minors also had elevated rates of ED utilization for mental health concerns (Table 6).

## Discussion

Despite frequent use of outpatient clinic services, LGBTQ patients in this study continued to experience higher rates of depression, suicidality, and increased dependence on the ED for crisis care. This is considered a negative indicator of care quality when used for non-emergent situations.

Previous research has shown that access to care for LGBTQ populations is a complex issue, and access to care within this study’s population is likely exacerbated by the rural and semi-rural setting. Previous studies have indicated that for LGBTQ populations, factors such as stigma, lack of specialized training for providers, and lack of insurance coverage are major contributors to barriers to accessing care [9]​. A recent publication documented both cruel and traumatic experiences of the LGBTQ population in healthcare settings, as well as avoidance of healthcare when needed due to stigma and discrimination [10]​. Other studies have shown that access to integrated interdisciplinary care that incorporates family-centered care, mental health, gender-affirmative care, and reproductive and sexual healthcare for LGBTQ populations has been found to improve health outcomes [7,11-14]. Furthermore, there is evidence showing that access to gender-affirming hormone treatment improves mental health outcomes in the TGD population [9,11-14]. Multiple studies have found that individuals treated with puberty blockers or cross-sex hormones experienced decreased depression and suicidality, improved psychological functioning, and body satisfaction. A prospective observational cohort study was conducted at an urban clinic among 104 trans and non-binary patients aged 13 to 20 years who were seeking gender-affirming care. In youth who had been treated with puberty blockers or cross-sex hormones, there was a decrease in both depression and suicidality by 60% and 73%, respectively. Even within the one-year time frame of this study, the authors demonstrated that access to puberty blockers or cross-sex hormones in a multidisciplinary gender-affirming setting was associated with improvements in mental health among transgender and non-binary youth. Access to these interventions is also associated with a decreased lifetime incidence of suicidal ideation among adults who received puberty blockers during adolescence [13]. Another study utilized longitudinal methodology to investigate whether 18 months of gender-affirming hormone treatment reduces symptoms of anxiety and depression in 1,271 transgender people. This study found that gender-affirming hormone therapy improved mental health in transgender individuals, especially among those with strong pre-existing social support. These results align with prior research showing that gender-affirming care and supportive environments enhance psychological well-being [6,12,14].

How to provide LGBTQ competent care is an area for further study, but previous reports have described several potential pathways. One such pathway is to improve the quality and quantity of LGBTQ-related training in medical education, with specific attention to primary care clinicians, to produce future medical providers who are culturally competent and able to deliver effective healthcare for LGBTQ individuals [15,16]. Additionally, multi-disciplinary LGBTQ health centers can provide comprehensive care for individuals across the LGBTQ community [15]. 

In addition to LGBTQ-focused health centers, broader health organizations can adopt strategies to reduce stigma and enhance access to essential services for LGBTQ individuals. System strategies might include expanding telehealth services for mental health and gender-affirming care and integrating behavioral health in primary care clinics. Establishing a culture of inclusivity and non-discrimination within the institution is essential. It encourages all staff and providers to collaboratively engage in building a space that welcomes people from diverse backgrounds [16]. In addition to institutional policies, the role of individual providers and staff is instrumental in providing an affirming healthcare experience to LGBTQ individuals. Every interaction, from front-desk encounters to clinical consultations, contributes to a positive healthcare experience for LGBTQ patients.

Limitations

This study is retrospective and cross-sectional in nature, which limits the ability to infer causal relationships or fully understand the factors driving higher ED utilization among LGBTQ patients. Associations can be described, but causality cannot be determined from this design. SOGI were identified based on self-reported data and ICD-10 codes, which are prone to incomplete reporting, underreporting, and coding inconsistencies, introducing potential misclassification bias. While statistical adjustments were performed, residual confounding from unmeasured variables (e.g., family support, community resources, or degree of social connectedness) may still impact mental health outcomes and were not accounted for in this analysis. While some misclassification is possible given reliance on EHR documentation, routine standardized data collection helps mitigate this risk. The gender distribution in our sample is consistent with national data, supporting generalizability. The data was analyzed in aggregate, which did not allow for capturing variation within specific subgroups of the LGBTQ population. Important subgroup differences (e.g., among racial or socioeconomic groups within LGBTQ cohorts) may, therefore, have been masked.

The study population is likely under-represented in this study for a number of reasons. The presence or absence of a gender dysphoria diagnosis may not fully capture the total TGD population. Not all individuals pursue or receive a formal diagnosis of gender dysphoria due to personal preferences, differences in clinical practices, or inconsistencies in medical documentation. This could lead to under-identification and misclassification bias. The study population only includes individuals who had at least one contact with the health system. LGBTQ individuals who entirely avoid healthcare due to stigma, discrimination, or other barriers were not included, which likely results in an underestimation of the true burden of depression, suicidality, and unmet mental health needs in this population. Mortality data, including individuals who died by suicide before having contact with the health system or during the study period, were not included. This omission may further underestimate the prevalence of severe mental health outcomes.

The study was conducted within a single health system serving a predominantly rural and semi-rural population. Structural barriers in these areas, including stigma, lack of LGBTQ-competent providers, and limited insurance coverage, may exacerbate inequities in access to care, reducing generalizability to more urban or diverse regions.

## Conclusions

This study adds to the existing literature, highlighting significant healthcare barriers and disparities faced by LGBTQ patients, particularly within a health system that primarily serves a rural patient population. This study identifies an underserved population with high emergency care utilization for mental health needs that could be better served in the outpatient setting. Better serving these needs in outpatient settings would theoretically lead to reductions in unnecessary ED utilization. The need for comprehensive outpatient care to support the mental health of LGBTQ populations, particularly for TGD youth, is clear and demonstrated. Access to LGBTQ friendly and competently trained providers, especially with gender-affirming practice, is needed to address the needs of this vulnerable population. In addition, the high burden of mental health diagnoses and crisis care utilization among the LGBTQ population necessitates focused attention from both healthcare teams and the broader health system. Further research may help identify healthcare system models and initiatives that effectively address these disparities for LGBTQ individuals.
